# The Relationship between Diet and the Occurrence of Depressive Symptoms in a Community Example with High Rates of Social Deprivation: A Cross-Sectional Study

**DOI:** 10.3390/nu15173778

**Published:** 2023-08-29

**Authors:** Grzegorz Józef Nowicki, Maciej Polak, Barbara Ślusarska, Karol Czernecki

**Affiliations:** 1 Department of Family and Geriatric Nursing, Faculty of Health Sciences, Medical University of Lublin, Staszica 6 Str., PL-20-081 Lublin, Poland; basiaslusarska@gmail.com (B.Ś.); karol@domaniolkow.pl (K.C.); 2 Department of Epidemiology and Population Studies, Jagiellonian University Medical College, Skawińska 8 Str., PL-31-066 Krakow, Poland; maciej.1.polak@uj.edu.pl

**Keywords:** depressive symptoms, diet, mental health, prevention, public health

## Abstract

Research suggests that various biological and psychosocial mechanisms are involved in the heterogeneous and complex relationship between dietary patterns and depressive symptoms. The occurrence of depressive symptoms is thought to be related to socioeconomic status (SES), with those with lower SES being more likely to experience persistent depression. The aim of the undertaken study was to investigate whether socioeconomic and health variables are associated with dietary assessment in a population with high rates of social deprivation and whether a relationship exists between dietary assessment and depressive symptoms (DS). The respondents’ nutrition was evaluated through a qualitative method, using the Perinumeric Periodic Table questionnaire by Starzyńska. At the same time, the prevalence of DS was assessed employing the Patient Health Questionnaire-9 (PHQ-9). In the DS screening (PHQ-9 ≥ 10), in the entire study population, the risk of DS was 16.1% (*n* = 605). In our entire study population, up to 61.2% (*n* = 2297) of the respondents exhibited poor dietary patterns. In the multivariate model, women with almost adequate or poor dietary assessment were 1.62 and 2.18 times more likely to score at least 10 on the PHQ-9 questionnaire, as compared to women whose dietary assessment was good or adequate. In conclusion, it was determined that sociodemographic variables affect nutritional habits. Women who lived in rural areas limited to a vocational education had significantly poorer diets. Moreover, men, younger men, smokers, and those without chronic diseases were characterized by a poorer dietary assessment. Additionally, women who had a better dietary assessment were significantly more likely to have lower scores on the questionnaire assessing the occurrence of DS (PHQ-9 10).

## 1. Introduction

In 2013, nutritional risk was shown to be responsible for more than a third of all deaths worldwide [1]. Therefore, according to the World Health Organisation (WHO), a change in dietary behaviour is essential [2]. Determinants of diet quality are multi-level and include intrapersonal, interpersonal, environmental, policy and culture-related factors. Several studies have demonstrated the link between diet, the living environment [3], and community socio-economic disadvantage [4]. Moreover, various epidemiological studies have shown that socio-economic status (SES) and demographic characteristics influence the prevalence of lifestyle risk factors [5]. Other authors indicate that higher SES is associated with better nutrition [6].

Depression is the fourth most serious illness in the world and one of the leading causes of self-injury and suicide [7]. The WHO has stated that more than 300 million people worldwide have a major depressive disorder, with depression being one of the leading mental health problems [8]. The root causes of depression have not been identified but are known to be related to various biological, genetic, psychological, and environmental factors [9]. Indeed, lifestyle factors are considered one of the crucial mediators of the pathophysiology associated with psychiatric disorders [10].

There is also growing evidence that diet may contribute to preventing depressive symptoms [11]. Several healthy eating patterns, including the Mediterranean (rich in vegetables, fruit and olive oil) [12], traditional Japanese (rich in fruits, vegetables, green tea and soy) [13] and traditional (rich in vegetables, fruits, fish and unprocessed meat) diets [14] are associated with a lower risk of depressive symptoms (DS). In contrast, however, there is a positive correlation between the occurrence of depressive symptoms with unhealthy dietary patterns, including the consumption of highly processed foods, sweets, fried foods, processed meat, refined cereals and high-fat dairy products [15], in other words, the typical Western, pro-inflammatory dietary pattern of today [16]. The results of the meta-analysis conducted by Tolkien et al. [17] suggest that a pro-inflammatory diet is associated with an increased likelihood of DS diagnosis, as those consuming a pro-inflammatory diet are 1.4 times more likely to develop DS when compared to those consuming meals rich in anti-inflammatory substances. People whose diets are rich in processed foods (consumed five times a week), such as fizzy drinks, fast food, sweets and red meat, are 39% more likely to have episodes of depression, compared to those whose diets were based on vegetables, fruit and milk [18].

Research suggests that a variety of biological mechanisms are involved in the heterogeneous and complex relationship between dietary patterns and DS, including reduced monoamine function, hypothalamic-pituitary-adrenal axis dysfunction, neuro-progression/brain plasticity, mitochondrial disorders [19], inflammatory processes mediated by cytokines, increased oxidative stress, immune reactions [20], inflammation of the immune system, intestinal dysbiosis and intestinal-cerebral reactions [19]. It is worth mentioning that the relationship between dietary patterns and mental health is complex and can be bidirectional. Indeed, dietary patterns may affect the occurrence of DS and depression may affect dietary patterns [21]. Furthermore, while most major depressive disorders cause an increase in appetite, body weight and body mass index values, resulting in chronic diseases, in contrast, the melancholic depressive disorder causes loss of appetite and weight loss [22]. Some dietary preferences associated with increased food intake induced by depression include fast foods, snacks, and low-quality and high-energy foods. In addition, people with severe depression are noted to reduce their intake of fruit, vegetables, fish, chicken, milk and cereals [23].

Given these findings, it is vital to study the impact of diet on depression in order to address the preventive and therapeutic potential of diet in dealing with depression. Based on this fact, it is necessary to identify strategies and methods that are effective in preventing depression and in alleviating its symptoms.

Socioeconomic inequality is thought to be related to depressive symptoms, where those with lower SES are more likely to have persistent depression [8]. On the other hand, living in a socioeconomically disadvantaged neighbourhood can also be a precursor to depression [24]. Epidemiological studies show that personal and socio-economic factors are associated with differences in the prevalence of depressive disorders. In addition, the prevalence of these factors and their distribution can vary over time, and are often linked to economic transitions or crises [25]. Poland, among Central and Eastern European countries, was the first to initiate political changes and the transition to a free market economy in 1989 [26]. This involved a gradual improvement in living conditions and quality of life, but it did not affect the whole of society equally [27]. Those with a university education were more mobile in the labour market and therefore had higher incomes. In contrast, this socioeconomic change was associated with the decline of heavy industry and a reduction in the demand for manual labour, which led to a deterioration in the economic situation of a large group of workers with low levels of education [28]. The poorer SES of these groups was also associated with increased pre-existing social problems and reduced access to health care [29]. The mechanisms described above may have contributed to regional variation in disease disorders, including depressive symptoms in local communities.

Little research has investigated this connection, particularly in the low SES local communities that had undergone the more negative impacts of the post-1989 socio-economic transition. As a consequence, the purpose of the study carried out was to assess whether socioeconomic and health variables are related to dietary assessment in a population that has a high rate of social deprivation and to evaluate the relationship between dietary assessment and the incidence of depressive symptoms through a case study of residents of the Janów Lubelski district in eastern Poland.

The residents of the Janów Lubelski district were chosen due to an analysis of several factors connected to the SES of the people living in this area before the survey was taken, which appeared very unfavourable in comparison with national indicators. For example, on the eve of the survey, a higher proportion of people with primary education in the Janów Lubelski district was revealed when juxtaposed with the general population (27.55% vs. 23.2%) [30]. Moreover, the unemployment rate was 15.6% among people of the productive working age group when it was set alongside the national unemployment rate of 14% [31]. In addition, the statistics regarding the number of people in the families that had received social assistance in the Janów Lubelski district was relatively high—as much as 14.6% of the entire population were on social assistance (when compared to the average in Lubelskie Voivodeship, which showed a figure of 9.4%) [32].

## 2. Materials and Methods

### 2.1. Study Design and Participants

From 14 June 2015 to 20 March 2016, a prevention and health promotion programme entitled “Take your health to heart” was conducted in the district of Janów Lubelski in the Lubelskie Voivodeship in eastern Poland. Data were collected during the implementation of this programme for scientific studies. The programme was financed under the PL 13, Reducing Social Inequalities in Health of the Norwegian Financial Mechanism 2009–2014, in Janów Lubelski District, and was made feasible because it covered local communities with high standardized mortality rates (SMRs). The selection of participating counties was based on the highest standardized mortality ratios (SMRs) between 2009 and 2011 for various categories, including malignant tumours, cardiovascular diseases, respiratory diseases, digestive diseases, external causes, and total mortality. Janów district ranked third on this list (SMR = 1.357) among all 38 counties in Poland, with the highest standardized cardiovascular mortality rates [33]. The programme targeted people between the ages of 35 and 64, and only individuals within this age group were allowed to participate.

In 2015, the population of the Janów Lubelski district was 47,500, out of which 18,827 were between the ages of 35 and 64. The recruitment process took place through the district and municipal local governments, with information disseminated in cooperating institutions within the Janów Lubelski district, such as churches, religious associations, workplaces, associations and public interest organizations. Invitations were also directly delivered via telephone to eligible individuals. To ensure equal and easy access to the programme, 15 points were set up in the Janów Lubelski district where respondents could enrol. These included 14 mobile points located across various places in the district and one stationary point established in the Municipal Hospital, which also served as the coordination centre for all activities.

Out of 4040 enrolled participants, the participation rate from the eligible population was 21.45%. Of all the participants, 421 people (10.42%) did not meet the inclusion criteria and were excluded from the study. The inclusion criteria for the study were: (1) age between 35 and 64, (2) no history of cardiovascular incident, (3) no known ischaemic heart disease, and (4) informed consent. The exclusion criteria were as follows: (1) a previous cardiovascular event (a heart attack or a stroke); (2) a diagnosis of ischemic heart disease; (3) pregnancy; (4) a lack of informed consent to participate in the study; (5) constant immobilisation; and (6) residing in a care home or prison. The subsequent analyses excluded 133 subjects with a history of a cardiovascular incident (myocardial infarction or stroke) and 288 subjects diagnosed with ischaemic heart disease. The final analysed group comprised 3752 people who met the inclusion criteria.

### 2.2. Data Collection

The data and anthropometric measurements were collected by a team of specially trained nurses. All participants completed a questionnaire survey and underwent anthropometric testing (measurements of body weight and height).

#### 2.2.1. Dietary Assessment

Respondents’ diets were evaluated using a qualitative method involving the Peri numeric Periodic Table questionnaire by Starzyńska [34]. The questionnaire comprised six questions related to the number of meals per day (4–5 vs. 3 vs. less), the number of meals with animal protein (in all meals vs. 75% of meals vs. in fewer meals); regularity of milk and milk products consumption (every day in 2 meals, as opposed to daily in at least 1 meal and on 50% of days in 2 meals vs. less frequently); regularity of wholemeal bread, groats and pulses consumption.

Respondents received points for their answers, and the total points determined their dietary assessment, classified as good, adequate, almost adequate, or poor. For each question, the respondent could receive 5 points, 3 points or 0 points, resulting in a maximum of 30 points for the whole survey. The final results were interpreted within the following categories: 30 points when eating well (good diet); 21–28 points when eating adequately (adequate diet); 12–20 points but without 0 scores—was defined as eating almost adequately (almost adequate diet); 11 points and below was characterized as poor diet. The questionnaire showed a high consistency of repeatability with a Cronbach’s alpha coefficient of 0.73. The high consistency of the tool was confirmed by Starzyńska, when confronted with the method of quantitative evaluation of menus (according to the amount of energy provided by the food ration and the content of protein, fat, carbohydrates, vitamins and minerals (using a computer program)) [35].

#### 2.2.2. Depressive Symptoms

The occurrence of depressive symptoms in the past 2 weeks was assessed using the Patient Health Questionnaire-9 (PHQ-9) [36]. The questionnaire was a self-report tool and consisted of nine questions marked by the respondents on a scale from 0 (‘not at all’) to 3 (‘almost every day’) how regularly they experienced such symptoms as anhedonia, a depressed mood, sleep disturbance, fatigue, appetite changes, low self-esteem, concentration problems, psychomotor disorders, and suicidal ideation. The nine items on the questionnaire corresponded to the nine diagnostic criteria for defining a significant period of depression according to the Diagnostic and Statistical Manual of Mental Disorders (DSM). The scale scores were calculated as the sum of 9 items, with a possible range of 0 to 27. The United States Preventive Services Task Force (USPSTF) and other organizations have advocated it for screening for DS in primary care and for screening for depression in the populace more generally. Still, the recommendations do not precisely indicate the scoring method [37]. A PHQ-9 score of at least 10 points indicates the risk of depressive symptoms. The PHQ-9 questionnaire demonstrated ideal internal consistency in the study group [38].

#### 2.2.3. Anthropometric Measurements

Height and weight measurements were taken for all subjects. Height was measured to the nearest 0.1 cm and weight without shoes or other clothing was measured with a platform scale to the nearest 0.1 kg. BMI was calculated as body weight (kg) divided by height squared in metres (kg/m^2^) and subjects were classified as: normal weight when BMI equals between 18.5 and 24.9 kg/m^2^; overweight when BMI equals between 25 and 29.99 kg/m^2^ and obese with BMI values ≥ 30 kg/m^2^ [39].

#### 2.2.4. Other Socioeconomic and Health Variables

Data on age, gender, place of residence, marital status, education, smoking status, cohabitation and comorbidities (doctor-diagnosed chronic diseases such as hypertension or diabetes or hypercholesterolemia) were collected using a standardized interview questionnaire.

Smoking status was defined as a non-smoker (if the respondent has never smoked tobacco or if the respondent quit at least one month before participating in the survey) and a smoker (if the respondent smokes at least 1 cigarette/day or if the last cigarette was smoked within the last month). 

The study participants were asked how much alcohol they had consumed in the year before the study, specifically, how frequently they consumed 1–2 standard doses of alcohol (SJA), assuming that 1 dose equalled 10 g of pure ethyl alcohol. The respondent could select from the following options: I do not drink alcohol (in the case of not drinking alcohol at all), less than once a month, once a month to once a week, and more than once a week.

### 2.3. Ethical Considerations

The study received ethical approval from the Bioethics Committee at the Medical University of Lublin (decision number: KE-0254/112/2014), and it was conducted following the principles of the Declaration of Helsinki. All participants provided written informed consent to participate in the study.

### 2.4. Statistical Analysis

Continuous data values were presented as mean (standard deviation [SD]) or median (interquartile range [IQR]), and qualitative data as the number and percentage. The Chi-squared test was used to compare qualitative data, while the Student’s *t*-test was used to compare the means between categories. Multivariable logistic regression assessed the relationships between dietary assessment and PHQ-9 ≥ 10. The choice of covariates included in the multivariable analysis was based on the literature and results of the univariate analysis. The logistic regression model results are presented as odds ratio (OR) with a 95% confidence interval (CI). The IBM SPSS Statistics for Windows, Version 28.0. (Armonk, NY, USA, IBM Corp) software was used for statistical analysis. A *p*-value less than 0.05 was considered significant for all tests.

## 3. Results

### 3.1. General Characteristics of Participants

Amongst the 3752 respondents, more than half were female (58.66%, *n* = 2201). The average age in the study group was 52 ± 8.1. The majority of the respondents lived in rural areas (66.9%, *n* = 2509), 88% (*n* = 3300) of all respondents were married. Most respondents were characterized by vocational education (37%, *n* = 1390), followed by secondary education (32.1%, *n* = 1204) and tertiary education (19.9%, *n* = 745). The fewest respondents completed primary education—11% (*n* = 413). Men, compared to women, were less educated (*p* < 0.001), were more likely to report smoking cigarettes (*p* < 0.001), were more likely to consume more significant amounts of alcohol (*p* < 0.001) and were less likely to have a normalized BMI (*p* < 0.001). Women were more likely to live alone than men (*p* = 0.03). The characteristics of the study group are presented in Table S1.

### 3.2. Dietary Assessment in the Study Group and the Relationship of Dietary Assessment to Selected Socioeconomic and Health Variables

Table 1 shows the distribution of answers to individual questions assessing the diet by gender strata. Compared to men, women are significantly more likely to declare eating 4–5 meals daily, more frequently consuming milk or cheese, vegetables and fruit and raw vegetables and fruit. They are also more likely to consume wholemeal bread, groats, and dry legumes. Only when asked about the presence of animal protein products in their meals are men more likely to declare that such products are found in all their meals.

The total score ranged between 0 and 30 points (median = 11; Q1 = 7, Q3 = 15), with only 10 respondents scoring the maximum number of points. Poor diet characterized 61.2% (*n* = 2297) of the respondents, including 69.4% (*n* = 1077) of all males and 55.4% (*n* = 1220) of all females amongst them. Moreover, 32.3% (*n* = 1211) of all respondents reported almost adequate diet, of which 26.4% (*n* = 409) were men and 36.4% (*n* = 802) were women. Additionally, 6.2% (*n* = 234) of the respondents were assessed as sustaining an adequate diet, amongst them 3.9% (61) were male and 7.9% (173) were female. Women were significantly more likely to exhibit a better dietary assessment than men (*p* < 0.001). Due to the small percentage of people with a dietary evaluation considered as good, the categories good and adequate were combined for further analysis and diet was analysed in three categories: ‘good/adequate’, ‘almost adequate’ and ‘poor’.

Table 2 shows the relationships between socioeconomic and health variables and dietary assessment. Place of residence and education were significantly associated with dietary assessment in women. Poor dietary assessment was significantly more frequent among women living in rural areas and those with vocational education. Among men, age, living alone, smoking and the presence of comorbidities (hypertension or diabetes or hypercholesterolaemia) revealed a significantly differentiated dietary assessment. A significantly higher prevalence of poorer dietary assessment was observed in younger men, those who smoked cigarettes and those without comorbidities.

### 3.3. Occurrence of Depressive Symptoms in the Study Group

The average PHQ-9 value in the sample was 6.4 ± 3.51 and was significantly higher in women (6.9 ± 3.5) than in men (5.8 ± 3.4) (*p* < 0.001). When screening for DS with the PHQ-9 questionnaire, the risk of depressive symptoms was observed in 16.1% (*n* = 605) of all respondents and this percentage was almost twice higher in women (19.6%, *n* = 431) than in men (11.2%, *n* = 174) (*p* < 0.001).

### 3.4. Relationship between Dietary Assessment and Risk of Depressive Symptoms in the Study Population and Gender Strata

**Figure 1:**
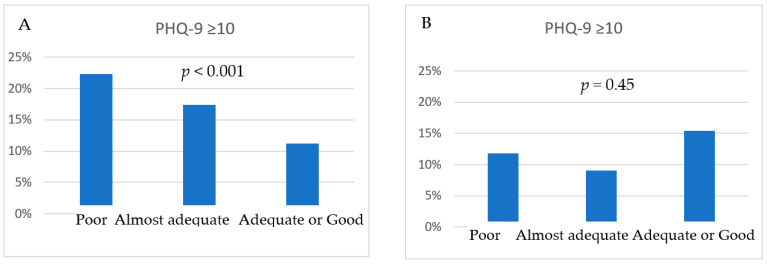
Comparison of participants with PHQ-9 ≥ 10 between categories of dietary assessment in women (**A**) and men (**B**).

Figure 1 presents the comparison regarding the proportion of DS participants between categories of diet assessment. In women, there was a significant decline in percentages of PHQ-9 ≥ 10 with an increase in the quality of diet. However, there was no association between PHQ-9 and dietary assessment in the case of men.

Table 3 shows the association between the PHQ-9 and dietary assessment. After adjusting for age, women with almost adequate or poor dietary assessment were 1.64- and 2.29-fold more likely to have DS, respectively, compared with women with a good dietary assessment. After adjusting for further covariates, the direction and strength of the association were similar. In men, no association was found between diet and PHQ-9.

## 4. Discussion

In this study, the relationship between socioeconomic variables and diet was investigated, and an assessment of the relationship between diet and the occurrence of depressive symptoms was conducted in a large sample of residents in a district in eastern Poland. This study stands out as one of the few to directly examine the impact of socioeconomic diet and the influence of diet on the risk of depressive symptoms in a local population characterized by a high social deprivation rate following systemic and economic changes, in this case, in Poland after 1989 (the data being derived in 2016).

In this study, female participants were characterized by better dietary regime. The dissimilarities between men and women in terms of diet portrayed in the literature [40,41] corresponded to the results outlined in our study. The authors observed that women were characterized by a significantly better dietary assessment than men. Wardle et al. [42] surveyed 19,298 respondents from 23 countries and established that women are more likely than men to give up high-fat foods, limit salt intake and are more likely to choose high-fibre meals. Enumerated differences in diet between genders are likely to be multifactorial. Moreover, the results of the authors’ study partly confirmed research already published [43] that concluded that better dietary assessment is associated with older age. The authors’ analysis reported that being a younger man is associated with a poorer diet, whereas this relationship is not observed among female participants. In the study population, this could be explained by the fact that male respondents when at an age when lifestyle diseases begin to be recognizable, choose or are pushed to change their diet to maintain the best possible health, whereas female respondents generally have a better dietary assessment over all age categories; therefore, age in our population among women might not have made a difference. This is confirmed by another observed relationship in the studied male population concerning better diet assessment amongst men burdened with chronic diseases. This is probably due to medical intervention in the form of advice. In addition, knowledge of healthy eating increases with age due to health education provided in, for example, community care or the media, and the positive feelings that result from healthy eating also increase [44].

In previously reported studies, education level was found to be strongly associated with a healthy diet [44,45]. Our study only observed a relationship between education level and better dietary assessment among women. A plausible explanation for the effect of higher education on better dietary intake is that the more educated base their choices on nutritional recommendations [46]. Moreover, people with higher education possess a higher income and can afford quality food [47]. Of note: in the traditional family model, women are more often responsible for procuring food items.

Among the other variables significantly influencing dietary assessment amongst female participants, rural inhabitants were noted to be characterized by worse dietary patterns. In contrast, among men, those living alone and smoking cigarettes demonstrate a worse dietary pattern. In the studied community, which is characterized by a high deprivation index, those living in rural areas had a lower income than those living in urban areas. Therefore, their SES was lower and their diet may have been of lower quality, in line with other studies [48]. A similar explanatory mechanism may apply to people living alone. Cigarette smoking-induced changes in the sensory system impair taste perception [49] and reduce olfactory abilities [50], hence, cigarette smokers choose foods with a more pronounced taste, e.g., with a higher salt content. Interestingly, BMI did not differ within the dietary assessment in both female and male groups. This finding raises the question that diet does not appear to influence body weight in our sample and conceivably, other factors may be of relevance concerning BMI values, such as portion size, total energy intake and physical activity levels. Such conclusions are similar to what was found in another study [51].

The prevalence of DS in studies in general populations using screening questionnaires varies. In a meta-research review by Levis et al. [52] covering meta-analyses of the prevalence of depressive symptoms published between January 2008 and December 2017, among the 69 meta-analyses included, 36 were based on screening tools and found a prevalence of 31% for depressive symptoms. In contrast, in our study, the prevalence of depressive symptoms was assessed using the PHQ-9 questionnaire and the incidence of the cut-off score (10) was 16.1%. The difference in the prevalence of depressive symptoms between our study and the research referred to above may be due to the fact that the aetiology of depression is associated with a number of risk factors that vary across communities. In addition, employing diverse screening questionnaires in studies may lead to an over- or underestimation of the results concerning the DS prevalence assessment, so further research in this area is required.

Diet quality is associated with the risk of depression, e.g., the quality of a mother’s diet influences her children’s mental health; also, the quality of diet influences the occurrence of depressive symptoms at any stage of life [53]. Gomes et al. [54] concluded that a poor diet doubles the likelihood of depressive symptoms in older women, compared to men. A meta-analysis found that diet quality, micronutrient intake and adherence to a Mediterranean diet may affect the incidence of depression [55]. Another study observed a strong association between adherence to a Mediterranean diet and the reduced risk of depression. Moreover, the severity of depressive symptoms is lower in people who consumed a lot of cereals and vegetables [56]. A further study concluded that the risk of depressive symptoms is higher in a group with a higher intake of refined sugar and sweets and a low intake of legumes, vegetables and fruit [57]. Ju et al. [58] also noted that the risk of depressive symptoms is higher in a group with a higher intake of refined sugar and sweets and a low intake of legumes, vegetables and fruit. The model found the same after accounting for confounding variables (age and energy intake). In other models, which included further confounding variables (age, energy intake, obesity, smoking, drinking alcohol, stress, frequency of eating out, eating breakfast and food security), however, it was observed that only in women and in the overall study population was the prevalence of depressive symptoms lower with high fruit and vegetable intake. 

In another study of 4969 individuals comprising different ethnic groups living in Amsterdam, Vermeulen et al. [59] found that a dietary pattern characterized by high amounts of chocolate, sweets and biscuits, red meat, sweetening, high-fat dairy products, fried foods and sauces was significantly associated with a higher prevalence of depressive symptoms. In multivariate models, an increase in scores against the dietary pattern resulted in a rise of 8 per cent in the number of points obtained on the PHQ-9 questionnaire. The results of the self-reported study showed that, as the quality of diet increased, the proportion of women who scored 10 points or more on the PHQ-9 Depression Symptom Assessment Questionnaire decreased. On the other hand, in multivariate analyses, women whose diet was assessed as merely adequate and adequate were 1.64 and 2.3 times more likely, respectively, to score 10 or more on the PHQ-9 questionnaire. Furthermore, after accounting for confounding variables, the direction and strength of the relationship were similar in the women’s group. In contrast, in men in multivariate analyses, there was no association between diet and depression symptom scores using the PHQ-9 questionnaire. This is explained by the fact that poor diet is associated with the prevalence of overweight and obesity, and obese women have higher serum leptin levels than obese men [60,61]. 

Leptin is an adipokine produced by adipose tissue with receptors in the hypothalamus which functions as an appetite suppressor. It also has a pro-inflammatory role [62]. Although it may have an antidepressant effect [63], high levels have been reported in obese individuals and patients with depression [60,64]. Therefore, higher leptin levels are thought to alter its physiological properties, thus promoting feelings of hunger in women and increasing inflammation, contributing to excessive weight gain and depressive symptoms. A second possible explanation is related to sex hormones. Excess body weight in women can reduce female sex hormones, which can cause a change in mood and the development of depressive symptoms [65]. In contrast, the prevalence of obesity in men that is associated with poor diet can affect testosterone levels, which is also associated with depressive disorders [66]. Other studies suggest that other factors may also influence the mechanisms involved in developing depressive symptoms in women and men, such as oestrogen-related activity or structural differences in the hippocampus [67]. 

The findings of our study highlight the importance of incorporating nutritional intervention into a biopsychosocial model for the prevention and treatment of mental disorders, particularly in rural communities with low socioeconomic status. The assessment of the occurrence of DS and assessment of diet could be conducted on a regular basis in primary care, where patients from local communities, such as the one under study, have the most frequent contact. Firstly, primary health care professionals should be trained in dietary assessment and nutrition education. Based on screening, patients should then be qualified to take part in public health initiatives aimed at improving their diet and lowering the incidence of DS. Interdisciplinary dietary interventions should concentrate on promoting friendly eating environments, such as dining with others or learning healthy eating habits through cooking workshops, in addition to educating people about healthy nutritional habits. Due to the low SES, such initiatives should also provide material assistance to their participants from a variety of public institutions, with the involvement of European Union bodies, in order to raise their SES. As a result, in such a holistic-based initiative, participants’ care should be provided by an interdisciplinary team that includes, inter alia, doctors, nurses, a dietician, a social worker, or an occupational therapist, and the entire program could take place as part of a coordinated care. The participants of such a program should receive nutritional assistance that enhances feelings of enjoyment and well-being during and after eating, that boosts self-efficacy in eating, improves mood, physical health and overall quality of life, or helps participants build supportive relationships in the community and receive social support that lowers the incidence of DS.

### The Strengths and Limitations

The presented study has strengths and weaknesses that deserve consideration. Firstly, it is a population-based study with a relatively large sample size. Secondly, we used the PHQ-9 questionnaire to assess the prevalence of depressive symptoms, which in our study population showed good reliability [38]. Thirdly, this is a study conducted in a group of respondents who are members of a local community characterized by a high rate of social deprivation reflected in a higher percentage of people with primary education, a higher unemployment rate and a higher rate of people using social assistance compared to national data. In addition, between 2009 and 2011, the local community surveyed was characterized by the highest mortality rate in the country due to cardiovascular diseases. Consequently, this is one of the few studies assessing the effect of diet on the risk of depressive symptoms in such a specific local community. Moreover, fourth, there is a lack of nationwide studies in Poland assessing the prevalence of mental health problems in the population and evaluating factors associated with SES. Our study, in some way, mitigates these deficiencies.

However, our survey has several limitations that also need to be considered. Firstly, since the respondents came from a single county in eastern Poland, the cross-sectional design of the survey makes it impossible to generalize the results to the whole population and limits the power of our study to causal inference since it only shows specific trends in relationships. More specifically, conversely, it may be the case that respondents with depressive symptoms may be eating more unhealthy foods, so that depression may be causing unhealthy eating. Therefore, prospective observational studies such as cohort or nested case-control studies are needed to confirm our findings. Secondly, we used a questionnaire to assess dietary intake that did not assess all nutrients, such as fresh or frozen fish or seafood intake. Because the consumption of fresh or frozen fish and seafood is not very popular among the Polish population, and the considerable price of these products makes it problematic for our study population characterized by low SES, this makes consumption of this category of food item to be irrelevant outside of coastal areas. Among food products in Poland, respondents in the nationwide survey stated that they most often consume bread and flour products, potatoes, pork, vegetables, sweets, and sweetened beverages. In contrast, less often, they consume fruit, veal, beef, milk and fish [68]. The used questionnaire was therefore adapted to the dietary trends in Poland. In addition, we employed a self-report questionnaire to assess diet based on the respondent’s memory. Respondents tend not to report unhealthy eating habits, which in turn may lead to a better assessment of diet than in the reality. Therefore, the observed relationship may be underestimated. Thirdly, the PHQ-9 questionnaire is used to screen and not to diagnose depression; therefore, the prevalence of DS in our study may have been overestimated. Fourth and finally, although we tried to include all confounding variables, other co-variables may not be included. However, without a doubt, our study contributes to the research on the association of diet with the occurrence of depressive symptoms in populations characterized by low SES.

## 5. Conclusions

In conclusion, in a study conducted in a local community with a high social deprivation rate, gender was the socio-demographic variable significantly associated with diet quality. Furthermore, women living in rural areas and with a vocational education had a significantly worse diet. In contrast, men, younger men, smokers, and those without chronic diseases were characterized by a poorer dietary assessment. Additionally, women who had a better dietary assessment were significantly more likely to have lower scores on the questionnaire assessing the occurrence of DS (PHQ-9 ≤ 10). In multivariate analysis after age adjustment, women with diets evaluated as almost adequate or poor were 1.64 times and 2.29 times more likely to have DS, respectively, as compared to women whose diet was assessed as good. In contrast, there was no correlation between diet and the prevalence of DS in men.

## Figures and Tables

**Table 1:** Raw results of the dietary assessment in the study group.

Variables	Female (*n* = 2201)	Male (*n* = 1551)	*p*
	*n* (%)	*n* (%)	
Number of meals per day:			
4–5 meals	1320 (60)	659 (41.8)	<0.001
3–2 meals	828 (37.6)	843 (54.4)	
Fewer than 3 meals	53 (2.4)	59 (3.8)	
Number of meals with products providing animal protein:			
In all meals	208 (9.5)	182 (11.7)	<0.001
In 75% of meals	942 (42.8)	843 (54.4)	
In fewer meals	1051 (47.8)	526 (33.9)	
Frequency of milk or cheese:			
In at least 2 meals	239 (10.9)	129 (8.3)	<0.001
Daily, at least in 1 meal and on 50% of days in 2 meals	802 (36.4)	422 (27.2)	
Less frequently	1160 (52.7)	1000 (64.5)	
Frequency of fruit and vegetables:			
Daily, at least in 3 meals	206 (9.4)	74 (4.8)	<0.001
Daily, in at least 2 meals	1073 (48.8)	581 (37.5)	
Less frequently	922 (41.9)	896 (57.8)	
Frequency of fruit vegetables in raw form:			
Daily	675 (30.7)	286 (18.4)	<0.001
In 75% of days	644 (29.3)	460 (29.7)	
Less frequently	882 (40.1)	805 (51.9)	
Frequency of wholemeal bread, cereals and dry legumes:			
Daily, at least one of the products listed	534 (24.3)	254 (16.4)	<0.001
On 75% of days, one of the products listed	763 (34.7)	513 (33.1)	
Less frequently	904 (41.1)	784 (50.5)	

**Table 2:** Relationship between selected socioeconomic and health variables and diet in the study group.

Variables	Dietary Assessment							
	Female (*n* = 2201)			*p*	Male (*n* = 1551)			*p*
	Poor Diet	Almost Adequate Diet	Adequate Diet/Good Diet		Poor Diet	Almost Adequate Diet	Adequate Diet/Good Diet	
Age [years]:	51 ± 8.02	52 ± 8.3	51 ± 8.2	0.17	52 ± 8.0	53 ± 8.0	53 ± 7.5	0.03
Place of living:								
Rural areas	858 (59.7)	477 (33.2)	103 (7.2)	<0.001	757 (70.7)	275 (25.7)	39 (3.6)	0.145
Urban areas	362 (47.4)	325 (42.6)	76 (10.0)		320 (66.7)	134 (27.9)	26 (5.4)	
Marital status:								
Married	1052 (54.9)	707 (36.9)	158 (8.2)	0.43	951 (68.8)	370 (26.8)	62 (4.5)	0.3
Single (bachelor/bachelorette)	76 (60.8)	43 (34.4)	6 (4.8)		111 (75.5)	34 (23.1)	2 (1.4)	
Widow/widower	92 (57.9)	52 (32.7)	15 (9.4)		15 (71.4)	5 (23.8)	1 (4.8)	
Education:								
Primary	130 (58.3)	75 (33.6)	18 (8.1)	0.002	129 (67.9)	54 (28.4)	7 (3.7)	0.27
Vocation	409 (61.3)	212 (31.8)	46 (6.9)		517 (71.5)	181 (25.0)	25 (3.5)	
Secondary	422 (54.4)	291 (37.5)	63 (8.1)		295 (68.9)	115 (26.9)	18 (4.2)	
University	259 (48.4)	224 (41.9)	52 (9.7)		136 (64.8)	59 (28.1)	15 (7.1)	
Smoking status:								
Yes	149 (59.6)	89 (35.6)	12 (4.8)	0.14	260 (75.4)	77 (22.3)	8 (2.3)	0.035
No	868 (54.1)	598 (37.3)	137 (8.5)		513 (66.5)	222 (28.8)	37 (4.8)	
In the past	203 (58.3)	115 (33.0)	30 (8.6)		304 (70.0)	110 (25.3)	20 (4.6)	
Living alone:								
Yes	1156 (55.4)	760 (36.5)	169 (8.1)	0.98	1031 (69.1)	401 (26.9)	61 (4.1)	0.04
No	64 (55.2)	42 (36.2)	10 (8.6)		46 (79.3)	8 (13.8)	4 (6.9)	
BMI [kg/m^2^]:								
Norm	363 (57.3)	209 (33.0)	61 (9.6)	0.2	197 (72.4)	68 (25.0)	7 (2.6)	0.31
Overweight	432 (54.4)	302 (38.0)	60 (7.6)		497 (69.4)	183 (25.6)	36 (5.0)	
Obese	418 (54.9)	287 (37.7)	57 (7.5)		381 (68.0)	158 (28.2)	21 (3.8)	
Comorbidities: ^#^								
Yes	344 (52.0)	263 (39.8)	54 (8.2)	0.088	277 (64.9)	128 (30.0)	22 (5.2)	0.045
No	876 (56.9)	539 (35.0)	125 (8.1)		800 (71.2)	281 (25.0)	43 (3.8)	

^#^ Comorbidities: hypertension and/or diabetes and/or hypercholesterolemia.

**Table 3:** Results of logistic regression examining the association between diet and prevalence of DS across gender strata.

Dietary Assessment		Women			Men		
		OR	95% CI	*p*	OR	95% CI	*p*
Model A	Good diet/Adequate diet	1			1		
	Almost adequate diet	1.64	(0.99–2.71)	0.05	0.54	(0.25–1.15)	0.11
	Poor diet	2.29	(1.41–3.72)	<0.001	0.76	(0.38–1.53)	0.44
Model B	Good diet/Adequate diet	1					
	Almost adequate diet	1.64	(0.99–2.71)	0.06	0.54	(0.25–1.16)	0.12
	Poor diet	2.16	1.33–3.53)	0.002	0.7	(0.34–1.43)	0.33
Model C	Good diet/Adequate diet	1					
	Almost adequate diet	1.62	(0.98–2.689)	0.002	0.54	(0.25–1.18)	0.12
	Poor diet	2.18	(1.39–3.56)	0.002	0.73	(0.35–1.50)	0.39

Model A: adjusted for age; Model B: adjusted for age, education, smoking status, place of residence and living alone; Model C: adjusted for age, education, smoking status, place of residence, living alone, BMI and comorbidities.

## Data Availability

The datasets used and/or analysed during the current study are available from the corresponding author on reasonable request.

## Supplementary Materials

The following supporting information can be downloaded at: https://www.mdpi.com/article/10.3390/nu15173778/s1, Table S1: Characteristics of the researched group according to their gender.
