# Supplementary material for: The Relationship between Diet and the Occurrence of Depressive Symptoms in a Community Example with High Rates of Social Deprivation: A Cross-Sectional Study
Source: Nutrients. 2023 Aug 29;15(17):3778. doi: 10.3390/nu15173778 (PMC10489963; doi:10.3390/nu15173778)
Supplement: Supplementary file 1 [file nutrients-15-03778-s001.zip › nutrients-2554937-supplementary.pdf]

nutrients

MDPI

Supplementary Materials

The Relationship Between Diet and the Occurrence of Depressive Symptoms in a Community Example With High Rates of Social Deprivation: A Cross-Sectional Study

Grzegorz Józef Nowicki, Maciej Polak, Barbara Ślusarska and Karol Czernecki

Table S1. Characteristics of the researched group according to their gender.

Variables	Female (n = 2,201)	Male (n = 1,551)	Total (n = 3,752)	p
Age [years]:	52 ± 8.2	52 ± 8.0	52 ± 8.1	0.19
Rural areas	1,438 (65.3)	1,071 (69.1)	2,509 (66.9)	0.02
Marital status:				
Married	1,917 (87.1)	1,383 (89.2)	3,300 (88.0)	< 0.001
Single (bachelor/bachelorette)	125 (5.7)	147 (9.5)	272 (7.2)	
Widow/widower	159 (7.2)	21 (1.4)	180 (4.8)	
Education:				
Primary	223 (10.1)	190 (12.3)	413 (11.0)	< 0.001
Vocation	667 (30.3)	723 (46.6)	1,390 (37.0)	
Secondary	776 (35.3)	428 (27.6)	1,204 (32.1)	
University	535 (24.3)	210 (13.5)	745 (19.9)	
Smoking status:				
Yes	250 (11.4)	345 (22.2)	595 (15.9)	< 0.001
No	1,951 (88.6)	1,206 (77.8)	3,157 (84.1)	
Alcohol consumption:				
No or less than once a month	2,140 (97.2)	1,205 (77.7)	3,345 (89.2)	< 0.001
Between once a month and once a week	42 (1.9)	195 (12.6)	237 (6.3)	
More than once a week	19 (0.9)	151 (9.7)	170 (4.5)	
Lives alone	116 (5.3)	58 (3.7)	174 (6.64)	0.03
BMI [kg/m^2]:				
Normal [18.5 – 24.99 kg/m^2]	633 (28.9)	272 (17.6)	905 (24.2)	< 0.001
Overweight [25 – 29.99 kg/m^2]	794 (36.3)	716 (46.3)	1,510 (40.4)	
Obesity [≥ 30 kg/m^2]	762 (34.8)	560 (36.2)	1,322 (35.4)	
Comorbidities (Yes)[#]	661 (30.0)	427 (27.5)	1,088 (29)	0.09
Patient Health Questionnaire (PHQ-9):				
Total score	6.9 ± 3.5	5.8 ± 3.4	6.4 ± 3.5	< 0.001
None (0-4)	574 (26.1)	579 (37.5)	1,153 (30.7)	< 0.01
Mild (5-9)	1,196 (54.3)	798 (51.5)	1,994 (53.1)	
Moderate (10-14)	366 (16.6)	150 (9.7)	516 (13.8)	
Moderately-Severe (15-19)	19 (1.2)	51 (2.3)	70 (1.9)	
Severe (20-27)	5 (0.36)	14 (0.64)	19 (0.55)	
PHQ-9 (≥ 10)	431 (19.6)	174 (11.2)	605 (16.1)	< 0.001

[#] Comorbidities: hypertension and/or diabetes and/or hypercholesterolemia.
